# An Optical Fiber Displacement Sensor Using RF Interrogation Technique

**DOI:** 10.3390/s16030277

**Published:** 2016-02-24

**Authors:** Hyeon-Ho Kim, Sang-Jin Choi, Keum Soo Jeon, Jae-Kyung Pan

**Affiliations:** 1 Department of Electrical Engineering and Smart Grid Research Center, Chonbuk National University, 567 Baekje-daero, Deokjin-gu, Jeonju 54896, Korea; khhjjh@jbnu.ac.kr (H.H.K.); sang_jin@jbnu.ac.kr (S.J.C.); 2 Wind Valley Co. Ltd., 4 Yulchonsan-daero, Haeryong-myeon, Suncheon 58025, Korea; ksjeon@windvalley.kr

**Keywords:** optical fiber displacement sensor, wavelength division multiplexing (WDM), Mach-Zehnder electro-optical modulator (MZ-EOM), radio frequency (RF), free spectral range (FSR)

## Abstract

We propose a novel non-contact optical fiber displacement sensor. It uses a radio frequency (RF) interrogation technique which is based on bidirectional modulation of a Mach-Zehnder electro-optical modulator (MZ-EOM). The displacement is measured from the free spectral range (FSR) which is determined by the dip frequencies of the modulated MZ-EOM transfer function. In experiments, the proposed sensor showed a sensitivity of 456 kHz/mm or 1.043 kHz/V in a measurement range of 7 mm. The displacement resolution of the proposed sensor depends on the linewidth and the power of the optical source. Resolution better than 0.05 μm would be achieved if an optical source which has a linewidth narrower than 1.5 nm and a received power larger than −36 dBm is used. Also, the multiplexing characteristic of the proposed sensor was experimentally validated.

## 1. Introduction

Fiber-optic displacement sensors are very attractive due to their numerous advantages such as light weight, compact size, fast response, high sensitivity, electromagnetic immunity, and multiplexing capability [1]. Fiber-optic in-line Mach-Zehnder interferometers (MZIs), which usually consist of two mode coupling elements, have been intensively investigated for various sensing applications such as refractive index, temperature, strain, and displacement measurements [2]. Displacement sensors based on in-line MZIs are simple, low-cost, and provide high-sensitivity [2,3,4]. Slow light was used to enhance the sensitivity of optical displacement sensors which were based on MZI and fiber-based lossy ring resonators [5,6].

Non-contact measuring has two chief methods, which are based on optical principles and electro-magnetic principles. A non-contacting magnetic coupling displacement sensor based on fiber Bragg grating (FBG) sensing technology was studied in [7,8]. Various non-contact optical sensing techniques that can be used to measure distances to objects, displacements, surface profiles, velocities and vibrations were previously discussed and compared [9]. Conventionally, fiber-optic displacement sensors can be classified into interferometry-based and intensity-based sensors. Most interferometry-based sensors require a highly coherent laser and a highly stable optical setup, they are not often used in commercial systems. Intensity-based fiber-optic sensors are known to measure short distances without contact in a cost-effective way, particularly if plastic optical fibers (POFs) are used. They, however, require a calibration to reduce the error due to illumination intensity variations, optical connection losses, variations of the target reflectivity, dust, dirt, and so on [10,11,12,13].

Recently, a differential reflective fiber-optic sensor for angular displacement measurement exploiting the fact that the distance between the emitting fiber and the receiving fiber shifts only the angular-power curve was presented [14]. A multichannel heterodyne non-contact fiber-optic vibrometer and an intensity-based fiber optic displacement sensor for micro- and electro-mechanical systems (MEMS) have also been introduced [15,16]. A Fabry-Perot interferometer built with a tapered optical fiber tip and a flat reflecting target with a simple non-contact displacement microfiber sensor using an adiabatic U-shaped tapered fiber have been proposed and demonstrated [17,18].

In this paper, we proposed and experimentally demonstrated a non-contact optical fiber displacement sensor. It uses a radio-frequency (RF) interrogation technique which is based on bidirectional modulation of a Mach-Zehnder electro-optical modulator (MZ-EOM) [19,20]. The proposed sensor is less sensitive to illumination intensity variations than intensity-based sensors. Also, the RF interrogation technique makes the system simpler than the interferometry-based sensors. The transfer function of the proposed system was obtained based on the microwave photonic technique and active detection. The displacement variation was calculated from the measured free spectral range (FSR). The performance limits and multiplexing capability for the proposed sensor were experimentally explored.

## 2. Sensor Structure and Operating Principle

**Figure 1:**
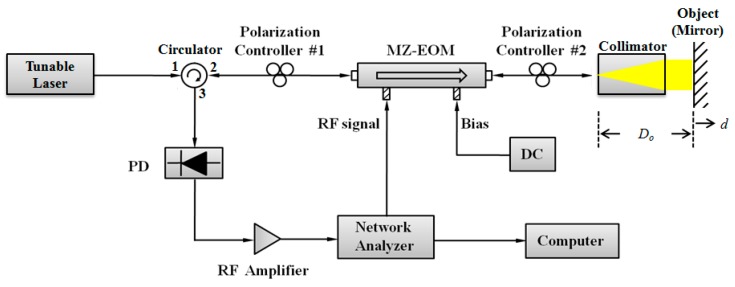
Schematic of the proposed non-contact optical fiber displacement sensor [21].

**Figure 2:**
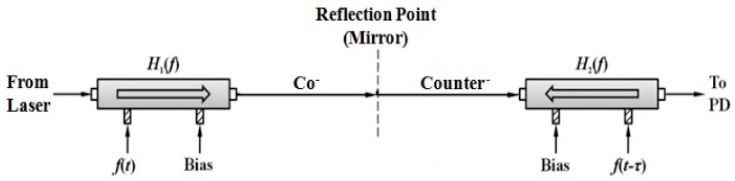
Equivalent model of bidirectional modulation with an MZ-EOM and a mirror.

A schematic diagram of the proposed non-contact optical fiber displacement sensor is shown in Figure 1. It consists of a tunable laser source (TLS), an MZ-EOM, an optical triplet collimator, a specular object, and a photodetector (PD). Light from TLS is modulated by the MZ-EOM which is driven by an RF signal. The modulated light is reflected back by a mirror and is modulated again by the same MZ-EOM. The bidirectionally modulated light is received by a PD and a subsequent network analyzer (NA). The bidirectional modulation in Figure 1 can be equivalently presented as two MZ-EOMs cascaded in series as in Figure 2 [19].

The travel time in terms of the displacement, τ(d), can be expressed as [21]:

(1) $$\tau \left(d\right)=\tau_{fiber}+\tau_{free}\left(d\right)=\frac{2L_{fiber}}{v_{g}}+\frac{2\left(D_{o}+d\right)}{c}$$

where $\tau_{fiber}$ is the travel time in optical fiber between the MZ-EOM and the collimator, $\tau_{free}\left(d\right)$ is the travel time in free space between the end of the optical fiber and the object, $L_{fiber}$ is the length of the optical fiber between the MZ-EOM and the collimator, $v_{g}$ is the group velocity of light in the optical fiber, $D_{o}$ is the length of free space between the end of the optical fiber and the object, d is the displacement from the initial point, and c is the velocity of light in free space. The time delay, ∆τ(d), which is the difference in the travel time between the initial point and the displacement, d, can be expressed as:

(2) $$∆\tau \left(d\right)=\tau \left(d\right)-\tau \left(0\right)=\frac{2d}{c}$$

From Equation (2), the time delay and the displacement have a linear relationship. The first MZ-EOM in Figure 2 lets the forward propagating light in Figure 1 experience co-propagating modulation by RF signal f(t). On the other hand, the second MZ-EOM in Figure 2 lets the back-reflected light in Figure 1 experience counter-propagating modulation caused by RF signal f(t−τ). Assuming that the modulation indices are very small and the bias is set to quadrature point, we can obtain an expression for the output optical power $P_{out}\left(t\right)$ at the PD as [19,20]:

(3) $$P_{out}\left(t\right)=\frac{P_{in}T_{D}}{4}\left[1+m_{1}H_{1}\left(f\right)cos\;2\pi ft\right]\;\cdot \left[1+m_{2}H_{2}\left(f\right)cos\;2\pi f\left(t-\tau \right)\right]$$

Here, $P_{in}$ is the input optical power, $T_{D}$ is the coupling and optical transmission losses of the structure, $m_{1}$ and $m_{2}$ are the modulation indices of co- and counter-propagating modulation, respectively, and $H_{1}\left(f\right)$ and $H_{2}\left(f\right)$ are the transfer functions of co- and counter-propagating modulation of the MZ-EOM, respectively.

The DC and harmonic components in Equation (3) are eliminated at the PD and the NA. Consequently, if we assume $m_{1}=m_{2}=m$, the total transfer function H(f) measured at the NA can be written as [19,20]:

(4) $$H\left(f\right)=A_{0}\left[H_{1}\left(f\right)+e^{-j2\pi f\tau }H_{2}\left(f\right)\right]$$

Here, $A_{0}=mRG_{m}P_{in}T_{D}/4$, R is the responsivity of the PD, and $G_{m}$ is the gain of the RF amplifier. The FSR is formed by dip frequencies in the transfer function and depends on the travel time in Equation (4). The FSR can be expressed as a function of displacement [21]:

(5) $$FSR_{d}=\frac{1}{\tau \left(d\right)}$$

Upon measuring the change in the FSR due to the displacement variation, the travel time and time delay can be calculated from Equations (1), (2) and (5).

## 3. Measurements and Results

### 3.1. Simulation Results

**Figure 3:**
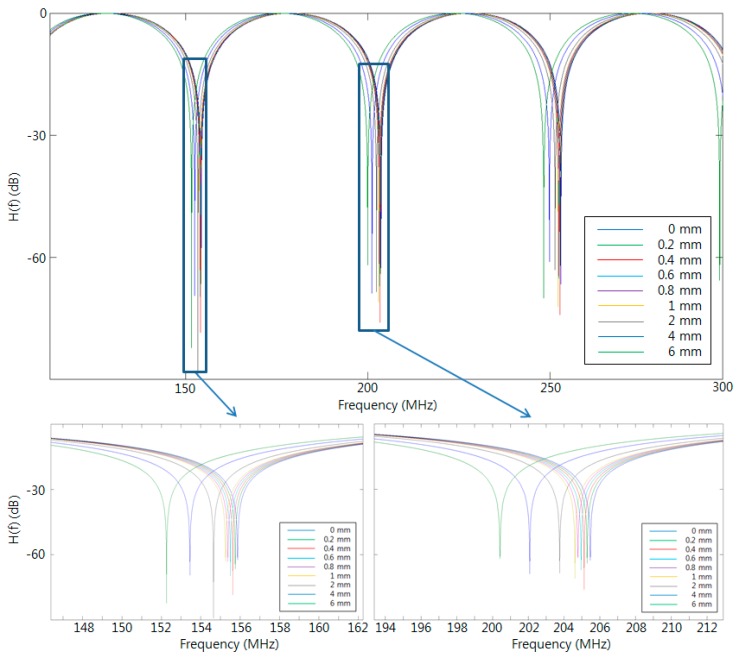
Calculated transfer function H(f) for nine displacements with $L_{fiber}$ of 1.5 m and $D_{o}$ of 75 mm.

Figure 3 shows the calculated transfer function H(f) for nine different displacements over the modulation frequency range of 100~300 MHz. We assumed an $L_{fiber}$ value of 1.5 m, a $D_{o}$ of 75 mm, and an optical fiber refractive index of 1.4416. As the displacement increases, the corresponding FSR decreases. The ability to distinguish displacement variation improves for shorter optical fiber.

**Figure 4:**
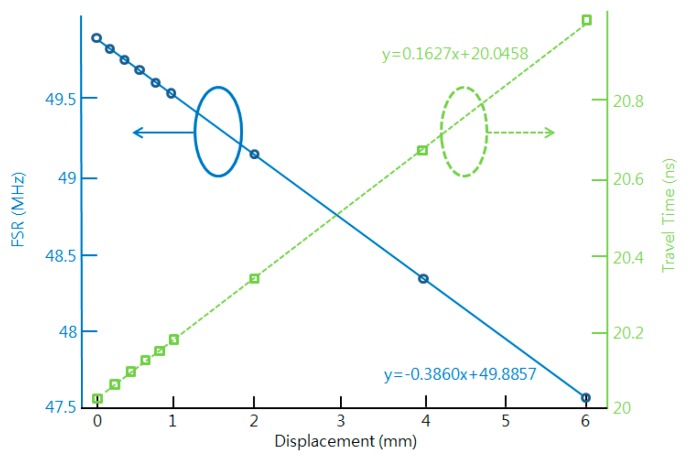
The FSR (solid blue line) and the travel time (dotted green line) as a function of the displacement variation.

Figure 4 shows the FSR and the travel time for nine displacements. The FSR and the time delay show a linear relationship according to the displacement with slopes of 386.0 kHz/mm and 162.7 ps/mm, respectively. The system performance, including the maximum measurement range and the resolution, can be further improved by using a shorter optical fiber between the MZ-EOM and mirror, an increased number of NA sampling points, and an optical source with a narrower linewidth and larger output power.

### 3.2. Experimental Results

**Figure 5:**
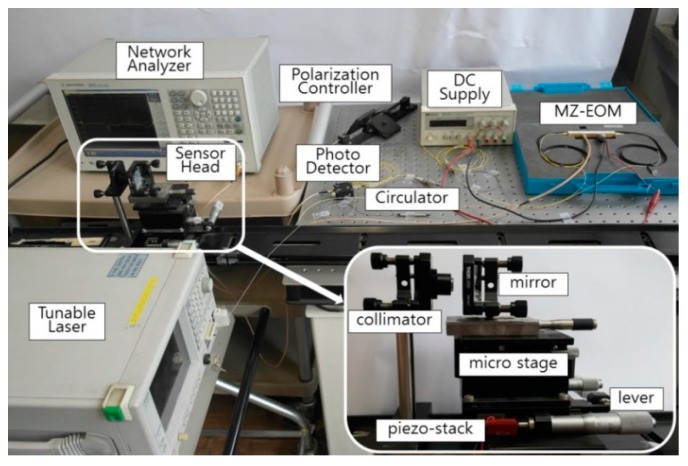
Experimental setup for the proposed optical fiber displacement sensor.

**Figure 6:**
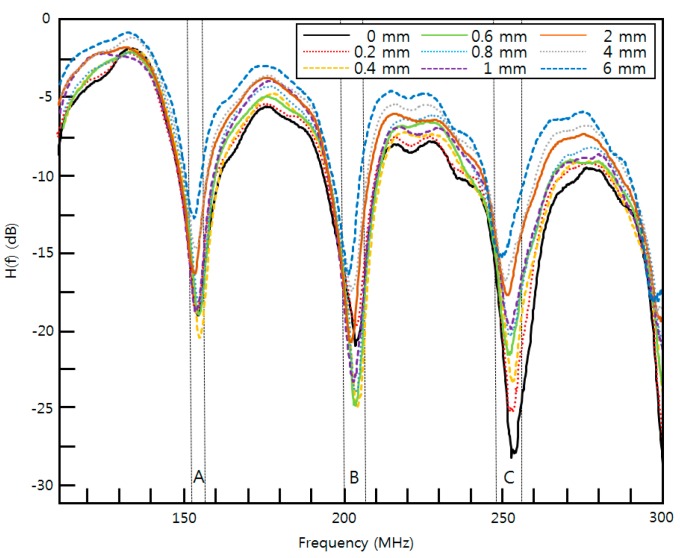
Measured transfer function H(f) with the proposed optical fiber displacement sensor for nine displacements.

The experimental setup of the proposed optical fiber displacement sensor is shown in Figure 5. The setup consists of a TLS (MG9638A, Anritsu, Tokyo, Japan) with an output power of 4 dBm, a circulator (6015-3-FC, Thorlabs, Newton, NJ, USA) of three ports, a polarization controller (973/579-7227, Thorlabs), a mirror (PF10-03-G01, Thorlabs), an MZ-EOM (MXAN-LN-10, Photline, Besançon, France) with a bandwidth of 10 GHz, a DC supply (E3630A, Hewlett Packard, Englewood, CO, USA), a triplet collimator (TC12FC-1550, Thorlabs) with a beam divergence of 0.101°, a PD (DET01CFC/M, Thorlabs) with a bandwidth of 1.2 GHz, and a network analyzer (NA) (N5230C, Agilent, Englewood, CO, USA) with an output RF signal of 1 dBm. The NA was set to sample 1601 points using an average factor of 100. We measured the transfer function, H(f), of the proposed sensor for nine displacements with an $L_{fiber}$ (PM 15-U25D, Corning, Corning, NY, USA) of 1.5 m, and a $D_{o}$ of 75 mm as shown in Figure 6. A micro-stage with a resolution of 10 μm and a piezo-stack with 10 μm displacement over 50 V (*i.e.*, 0.2 μm/V) were used to vary the displacement with sub-micron resolution. Figure 6 is one of 30 sets of experimental results obtained with the proposed optical fiber displacement sensor at 0, 0.2, 0.4, 0.6, 0.8, 1, 2, 4, and 6 mm in the frequency range of 100~300 MHz.

**Figure 7:**
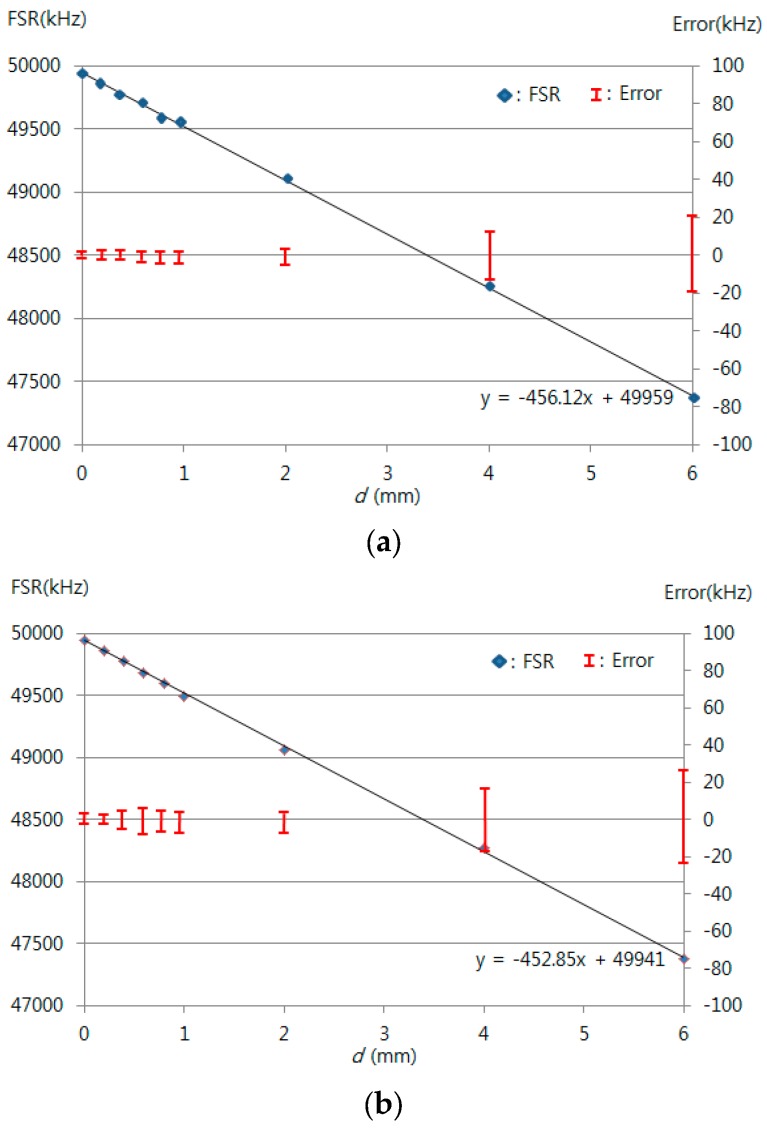
Measured FSR for the displacement variation using a micro-stage (resolution of 10 μm) between (**a**) dip A and dip B; (**b**) dip B and dip C.

**Figure 8:**
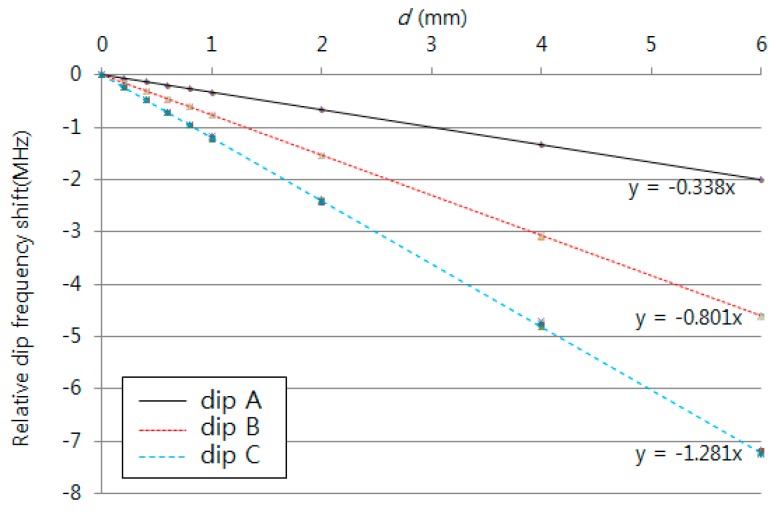
Measured dip frequency shift for the displacement variation using a micro-stage.

Figure 7 shows the FSRs obtained with dips A and B and dips B and C using the micro-stage. These regions showed sensitivities of 456 kHz/mm and 453 kHz/mm, respectively. The average and maximum relative errors of the FSRs are 0.005% and 0.031% with dips A and B and 0.032% and 0.059% with dips B and C. The sensitivities within the two ranges were very similar to one another. The relative errors increased with increasing measurement frequency. Figure 8 shows the measured dip frequency shifts for displacements of 0, 0.2, 0.4, 0.6, 0.8, 1, 2, 4, and 6 mm for dips A, B, and C, which have sensitivities of 338, 801, and 1281 kHz/mm, respectively. The average and maximum relative errors of the dip frequency shifts were 0.001% and 0.002% for dip A, 0.031% and 0.040% for dip B, and 0.062% and 0.089% for dip C. The sensitivity and the measurement error increased as the measurement frequency range increased. Thus, the sensitivity and measurement errors in dip C were the largest among the three dips.

**Figure 9:**
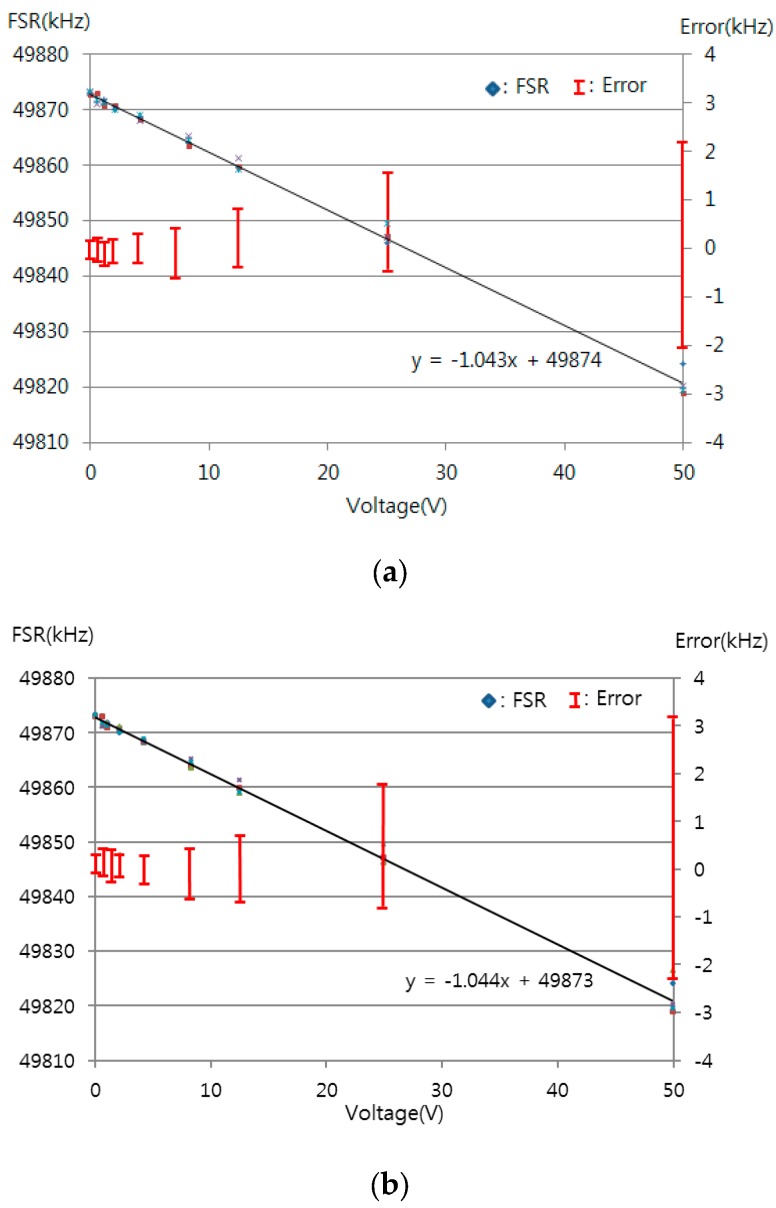
Measured FSR for the displacement variation using piezo-stacks (10 μm displacement over 50 V) between (**a**) dip A and dip B; (**b**) dip B and dip C.

**Figure 10:**
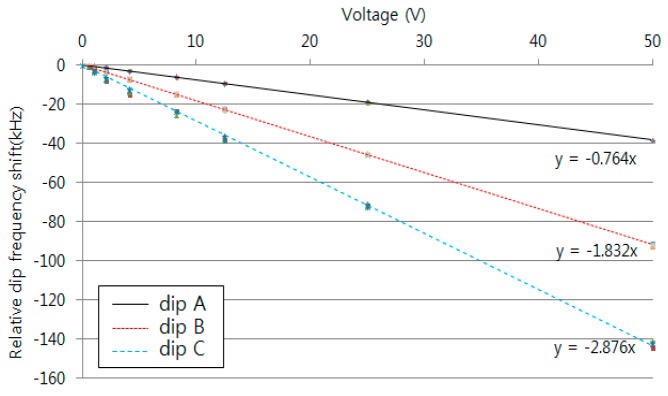
Measured dip frequency shift for the displacement variation using piezo-stacks.

Figure 9 shows the FSRs obtained for dips A and B and dips B and C using piezo-stacks with a resolution of about 10 μm over 50 V to precisely measure displacement. The FSRs had sensitivities of 1.043 kHz/V and 1.044 kHz/V, respectively. The average and maximum relative errors of the FSRs were 0.072% and 0.293% for dips A and B and 0.073% and 0.302% for dips B and C. The slope was similar to that obtained from the micro-stage, and the resolution was about 240 nm. Figure 10 shows the measured dip frequency shift with dips A, B, and C using piezo-stacks. These dips showed sensitivities of 0.764, 1.832 and 2.876 kHz/V, respectively. The average and maximum relative errors of the dip frequency shifts were 0.002% and 0.021% for dip A, 0.035% and 0.118% for dip B, and 0.088% and 0.274% for dip C.

### 3.3. Performance Limits

**Figure 11:**
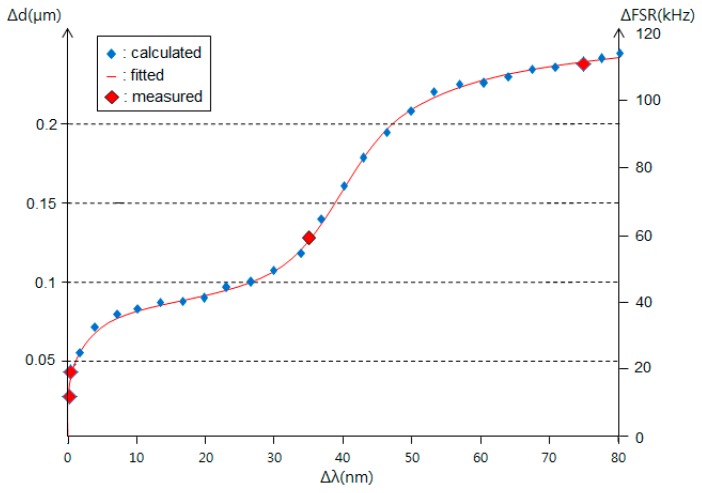
The displacement differential according to the input optical source linewidth.

To explore the proposed sensor performance limits, we considered the input optical source linewidth and the received optical power at PD. Figure 11 shows the FSR differential, ∆FSR, and displacement differential, ∆d, as a function of the input optical source linewidth, ∆λ. The small blue rhombuses, the red solid line, the large red rhombuses in Figure 11 are the calculated, the curve fitted, and the measured values, respectively. Four measured values using a TLS (MG9638A, Anritsu) with ∆λ of 0.2 nm, an arrayed waveguide gratings (AWG, AWG-G-50G-16-002-C-PN, POINTek, Anaheim, CA, USA) with ∆λ of 0.4 nm, a superluminescent LED (SLED, DL-BZ1-CS5403A-FP, DenseLight, Singapore) with ∆λ of 35 nm, and a broadband light source (BLS, OFB-BFB-21, LiComm, Yongin-si, Korea) with ∆λ of 75 nm were in good agreement with the calculated values. These data show that the resolution of the sensor depends on the input optical source linewidth, which provides a sensor resolution of 0.05 μm or less with an input optical source linewidth of 1.5 nm or less.

**Figure 12:**
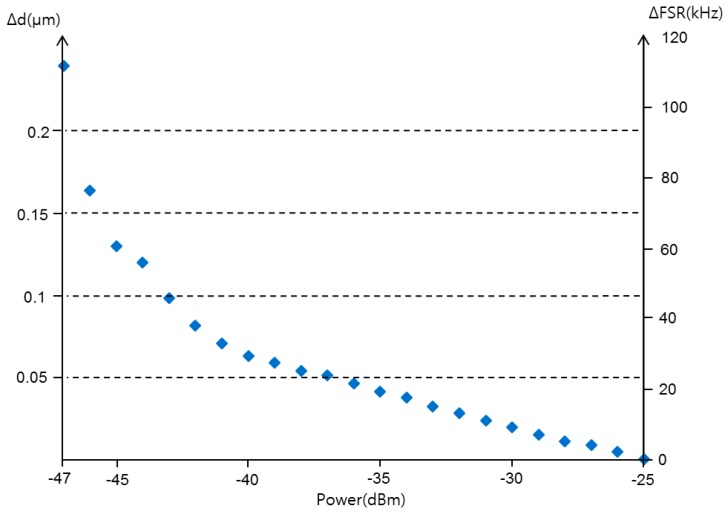
The displacement differential as a function of the received optical power at PD.

In order to find out if the sensor performance is limited by the received optical power at PD, we measured H(f) and calculated the corresponding ∆FSR and ∆d according to the varying received optical power, which are shown in Figure 12. As the received optical power decreased, the dips in H(f) became weaker and the corresponding ∆FSR and ∆d increased. The optical power should therefore be more than −36 dBm for a sensor with a resolution of 0.05 μm.

### 3.4. Optical Power Budget

For a given set of components and system requirements, we carried out a power budget analysis to determine whether the operating range of the proposed sensor meets the calculated power margin. The proposed sensor loss budget simply considers the total optical power loss $P_{T}$ that is allowed between the TLS and the PD shown in Figure 1. This loss was primarily allocated to component loss, connector loss, the loss of the circulator and mirror, and the optical power margin. Thus, if $P_{in}$ is the optical power emerging from the TLS, and if $P_{R}$ is the PD sensitivity, then:

(6) $$P_{T}=P_{in}-P_{R}=K_{circ1-2}+2K_{PC}+K_{MZ-forward}+K_{MZ-backward}+K_{circ2-3}+K_{coll+mirror}+optical\;power\;margin$$

**Figure 13:**
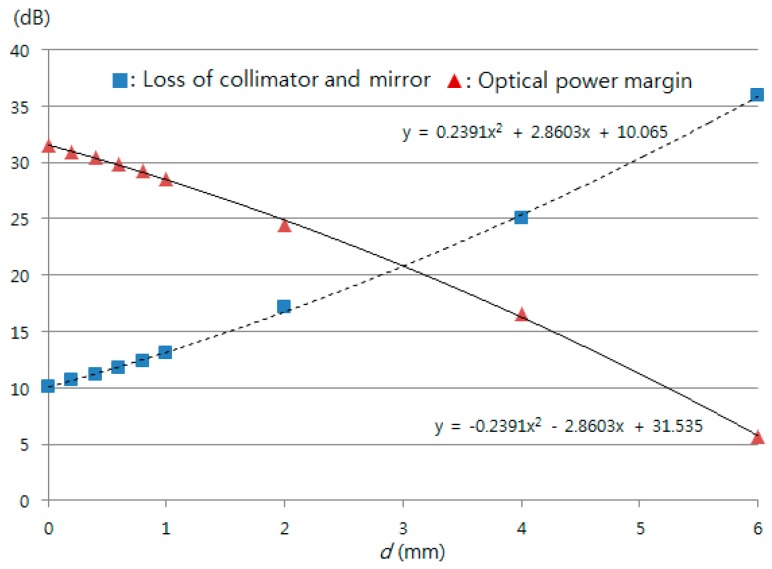
The proposed sensor optical power margin according to the displacement.

In our experiments, $P_{in}$ = 2 dBm and $P_{R}$ is –47 dBm. The losses of the components used in the experiments were a circulator loss (ports 1 and 2), $K_{circ1-2}$, of 1 dB, a polarization controller loss, $K_{PC}$, of 0.2 dB, MZ-EOM loss (forward), $K_{MZ-forward}$, of 2.8 dB, MZ-EOM loss (backward) loss, $K_{MZ-backward}$, of 2.1 dB, and a circulator loss (ports 2 and 3), $K_{circ2-3}$, of 1.1 dB. The losses of the circulator and mirror were 10.1, 10.7, 11.2, 11.8, 12.4, 13.1, 17.1, 25.1, and 35.9 dB for displacements of 0, 0.2, 0.4, 0.6, 0.8, 1.0, 2.0, 4.0, and 6.0 mm, respectively, as shown in Figure 13. Figure 13 also shows the measured losses of the collimator and mirror for the above nine displacements and the optical power margin corresponding to the measured losses. A measurement range of about 7 mm for a given set of components and system requirements was estimated since the optical power margin was 5.7 dB at a displacement of 6 mm.

### 3.5. Multiplexing Characteristic

**Figure 14:**
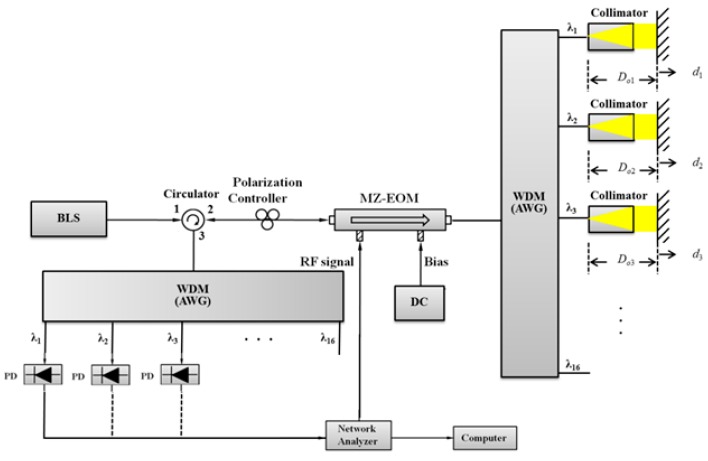
Experimental setup to validate the proposed sensor multiplexing characteristic.

**Figure 15:**
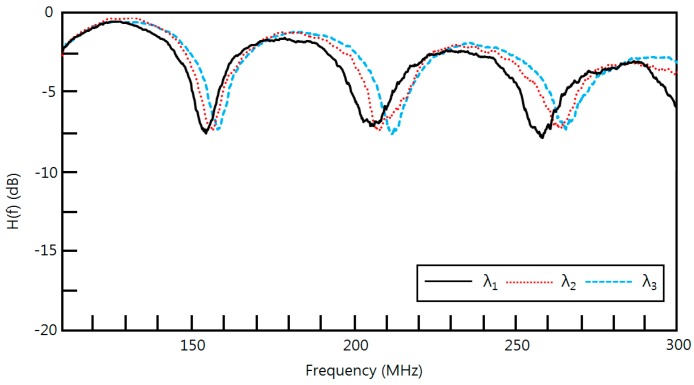
Measured transfer functions H(f) using three different wavelengths of BLS in Figure 14.

In order to confirm the multiplexing characteristic of the proposed sensor, we implemented an experimental setup shown in Figure 14. This setup consists of two AWGs (AWG-G-50G-16-002-C-PN, POINTek) with a channel spacing of 0.4 nm as a wavelength division multiplexer (WDM) and a wavelength division demultiplexer. A BLS (OFB-BFB-21, LiComm) with a wavelength of 1528~1603 nm and an output power of 21 dBm was used to obtain the experimental results shown in Figure 15. Figure 15 is one of 10 sets of experimental results obtained with the experimental setup at different distances of $D_{o1}$, $D_{o2}$, and $D_{o3}$ in the frequency range of 100~300 MHz.

The measured H(f) for three wavelengths of $\lambda_{1}$ (1552.3 nm), $\lambda_{2}$ (1552.7 nm), and $\lambda_{3}$ (1553.1 nm) showed three dip frequencies. The obtained FSRs from the first dip and second dip in H(f) for $\lambda_{1}$, $\lambda_{2}$, and $\lambda_{3}$ are 52.7147, 53.8346 and 54.9628 MHz, respectively. Their maximum relative errors were less than 0. 623%. We could achieve better resolution using a larger input optical source power.

## 4. Conclusions

A non-contact optical fiber displacement sensor using a radio frequency (RF) interrogation technique has been proposed. It is based on bidirectional modulation of an MZ-EOM. The transfer function of the proposed system was obtained using the microwave photonic technique and active detection. The relationship between the FSR and the displacement variation for the proposed scheme was given. FSRs have been presented via both simulation and experiments as a function of the displacement variation. As the displacement changes from 0 to 6 mm, the FSR and the travel time change from 49.8734 MHz to 47.2684 MHz and from 20.0507 ns to 21.1558 ns, respectively. Experiments showed that the proposed sensor provided a sensitivity of 456 kHz/mm or 1.043 kHz/V in a measurement range of 7 mm. Also, the measured dip frequency shifts have been presented as a function of the displacement variation. The proposed sensor showed a resolution of less than 0.05 μm for an optical source linewidth of narrower than 1.5 nm and a received power larger than −36 dBm. These values depended on the linewidth and the power of the input optical source. Finally, we confirmed the multiplexing characteristic of the proposed sensor through experiments using two AWGs.
